# Perceived Psychologically Controlling Teaching and Foreign Language Learning Engagement: Indirect Associations Through Foreign Language Anxiety and the Moderating Role of L2 Grit

**DOI:** 10.3390/bs16091662

**Published:** 2026-09-16

**Authors:** Xin Huang, Yi (Esther) Su

**Affiliations:** Language and Cognitive Development Lab, School of Foreign Languages, Central South University, Changsha 410083, China

**Keywords:** foreign language learning engagement, perceived psychologically controlling teaching, foreign language anxiety, L2 grit, psychological network analysis

## Abstract

Foreign language learning engagement (FLLE) is central to sustained classroom involvement, yet little is known about how perceived psychologically controlling teaching (PPCT), foreign language anxiety (FLA), and L2 grit are jointly associated with FLLE among Chinese senior high school students. This study combined structural equation modeling (SEM) and psychological network analysis (PNA) in 285 Chinese EFL learners. SEM tested a moderated indirect association model, whereas PNA estimated conditional associations across FLLE dimensions. PPCT was positively associated with FLA and negatively associated with FLLE, with a significant indirect association through FLA. L2 grit was positively associated with FLLE but did not attenuate the negative association between FLA and FLLE. Instead, the negative association between FLA and FLLE became stronger at higher levels of grit. Dimension-specific sensitivity analyses indicated that this conditional pattern was heterogeneous across engagement dimensions, with the most consistent evidence observed for emotional engagement. PNA revealed selective dimension-level connections among PPCT, FLA, L2 grit, and engagement dimensions, with emotional engagement showing the highest expected influence. These findings suggest that higher L2 grit does not necessarily buffer the negative association between FLA and engagement and highlight the importance of reducing psychologically controlling teaching and anxiety-provoking classroom experiences.

## 1. Introduction

Foreign language learning engagement (FLLE) is an important construct in educational psychology and second language acquisition. It reflects students’ multifaceted investment in the behavioral, cognitive, emotional, and agentic aspects of classroom learning and is closely associated with academic performance (Hiver et al., 2024; Sadoughi & Hejazi, 2023). In EFL settings, where language development depends on sustained effort and active classroom involvement, understanding what shapes FLLE is particularly important.

This issue is particularly relevant in Chinese senior high school EFL classrooms. English learning is embedded in a highly evaluative and examination-oriented environment, while teachers play a central role in organizing classroom activities, evaluating performance, and shaping students’ opportunities to participate (Wang & Liu, 2022; Zhang & Hu, 2025). Under these conditions, students’ engagement may be especially sensitive to how they perceive teachers’ instructional and interpersonal practices. Examining these perceptions may therefore provide important insight into why students differ not only in whether they participate, but also in the quality and depth of their involvement in language learning.

Existing EFL research has predominantly focused on supportive teacher practices, including autonomy support, teacher support, care, immediacy, and rapport (Han, 2021; Sadoughi & Hejazi, 2021; Zhang & Hu, 2025). Comparatively less attention has been paid to perceived psychologically controlling teaching (PPCT), which reflects students’ perceptions of pressuring interpersonal practices intended to secure compliance (Soenens et al., 2012). Although controlling teaching has been examined in relation to agentic engagement among university EFL learners (Jiang & Zhang, 2021), much less is known about how PPCT is associated with the broader behavioral, cognitive, emotional, and agentic dimensions of FLLE, particularly among senior high school students.

A fuller understanding of this association also requires attention to the psychological processes and learner characteristics that may shape students’ responses to controlling classroom experiences. Foreign language anxiety (FLA) represents a particularly relevant emotional experience in language classrooms, whereas L2 grit reflects learners’ tendency to sustain effort and interest in pursuing long-term language-learning goals (Horwitz et al., 1986; Teimouri et al., 2022). Yet these factors have rarely been considered together in explaining how PPCT is associated with multidimensional FLLE. Addressing this gap requires examining both the overall relationships among these constructs and their finer-grained associations across different dimensions of engagement. 

Accordingly, this study examines how PPCT is associated with multidimensional FLLE among Chinese senior high school EFL learners, with FLA considered as a variable involved in the indirect association and L2 grit as a possible moderator of the FLA–FLLE association. It further considers these relationships at both the overall construct level and across the behavioral, cognitive, emotional, and agentic dimensions of engagement. By doing so, the study seeks to provide a context-sensitive account of how perceived teaching practices, language-specific emotional experiences, and learner characteristics are associated with multidimensional engagement in Chinese senior high school EFL classrooms.

### 1.1. Psychologically Controlling Teaching and Foreign Language Learning Engagement

Psychologically controlling teaching (PCT) refers to teachers’ use of intrusive practices to pressure students to think, feel, or behave in prescribed ways, often through guilt induction, shaming, expressions of disappointment, or conditional approval (Soenens et al., 2012). The present study focuses on students’ perceptions of these practices, hereafter referred to as perceived psychologically controlling teaching (PPCT). Unlike structured guidance or appropriate classroom discipline, which organizes students’ behavior through clear expectations and guidance, PCT seeks compliance by exerting pressure on students’ thoughts, feelings, or sense of self-worth. From an SDT perspective, such practices may frustrate the needs for autonomy, competence, and relatedness by creating experiences of pressure, inadequacy, and conditional acceptance (Ryan & Deci, 2020; Soenens et al., 2012). Importantly, outward compliance does not necessarily indicate meaningful engagement. Students may follow classroom demands without investing the effort, cognitive involvement, emotional connection, or initiative that characterize deeper engagement (Reeve et al., 2025). Given the multidimensional nature of FLLE, the association with PPCT may therefore differ across behavioral, cognitive, emotional, and agentic engagement.

Empirical evidence generally supports a negative association between controlling teaching and student engagement. Early studies linked controlling teacher behavior to lower effortful participation and poorer self-regulated learning (Assor et al., 2005; Soenens et al., 2012). More recent research has extended this pattern by showing that controlling teaching is associated with maladaptive coping responses and less adaptive motivational and engagement outcomes (Flamant et al., 2023). Recent longitudinal evidence from Chinese middle schools is particularly relevant. Controlling teaching was associated with lower subsequent behavioral and cognitive engagement over one school year, suggesting that the negative pattern is not confined to Western educational settings or cross-sectional data (Yue et al., 2026). Within EFL education, Jiang and Zhang (2021) likewise found that perceived controlling teaching was negatively related to agentic engagement among Chinese university learners. Their study, however, focused on university students and examined agentic engagement rather than the broader multidimensional structure of FLLE.

Taken together, recent research has strengthened the evidence linking controlling teaching with poorer engagement in general education, including among Chinese secondary-school learners. Evidence from EFL classrooms remains much more limited, particularly at the senior high school level. It is therefore unclear whether PPCT is associated with overall FLLE among Chinese senior high school learners and whether its conditional associations differ across behavioral, cognitive, emotional, and agentic engagement. Examining these dimension-specific associations is especially relevant in examination-oriented EFL classrooms, where observable participation and task completion may coexist with quite different levels of cognitive, emotional, and agentic involvement.

### 1.2. The Relationship Among Psychologically Controlling Teaching, Foreign Language Anxiety, and Foreign Language Learning Engagement

Foreign language anxiety (FLA) is a language-specific emotion involving tension, apprehension, and worry during foreign language learning and use, especially in communicative and evaluative situations (Horwitz et al., 1986). From an SDT perspective, PCT can frustrate students’ autonomy and competence by limiting volitional action and undermining their sense of effectiveness (Ryan & Deci, 2020). These experiences may also reduce students’ perceived control over learning activities and outcomes. This provides a link to control-value theory (CVT), which identifies control and value appraisals as key antecedents of achievement emotions. Anxiety is especially likely when important learning activities or outcomes are perceived as difficult to control (Pekrun, 2006, 2024). Thus, PPCT may be associated with greater FLA partly by reducing students’ perceived control over language learning and evaluation.

Empirical evidence provides converging support for this account. Directly controlling teacher behaviors have been associated with greater anxiety (Assor et al., 2005), while perceived controlling teaching has been linked to fear of negative evaluation (Salazar-Ayala et al., 2021) and, through need frustration, to negative affect among Chinese secondary-school students (J. Liu et al., 2017). Longitudinal evidence further associates controlling teaching with fear of failure, contingent self-worth, and challenge avoidance through need frustration (Bartholomew et al., 2018). In addition, recent research with Chinese high school EFL learners showed that classroom environments were related to anxiety partly through students’ perceived control, with lower control associated with greater anxiety (Qian & Huang, 2026). Together, these findings suggest that controlling classroom experiences are associated with anxiety and closely related emotional vulnerabilities, while their relationship with language-specific anxiety remains underexamined.

Evidence linking FLA to engagement has emerged across different language learning settings. In UK university settings, O’Reilly and García-Castro (2022) found that greater FLA was associated with lower online engagement. Research in Türkiye has also reported a negative relationship between engagement in English use and L2 anxiety, although the direction of prediction differed from the present model (Uztosun & Kök, 2024). Studies conducted in Chinese EFL contexts have linked greater anxiety with lower overall engagement among university learners, both directly and through classroom relational or emotion regulation processes (X. Li et al., 2024; M. Li, 2026; Chen & Guo, 2026). At the dimension level, the pattern is less uniform. Among Chinese high school EFL learners, H. Liu et al. (2025) found that English learning anxiety was positively associated with behavioral engagement but showed no significant direct association with agentic engagement. Taken together, the literature suggests that anxiety and engagement are related across different language learning contexts, while the form of this relationship varies with educational level, mode of instruction, and the specific dimension of engagement examined. 

### 1.3. The Relationship Among L2 Grit, Foreign Language Anxiety, and Foreign Language Learning Engagement

L2 grit refers to learners’ sustained perseverance and interest in pursuing long-term language learning goals (Teimouri et al., 2022). Research on this construct has been conducted across several EFL settings. Early work on L2 grit was carried out in Iranian EFL contexts, and subsequent studies extended this line of research to Chinese EFL learners. Studies in China have linked L2 grit with stronger classroom engagement (Jin, 2024). Research in Iran and Russia has also linked grit with online engagement, self-efficacy, achievement, and lower anxiety (Sudina & Plonsky, 2021; Derakhshan & Fathi, 2023). This body of evidence indicates that L2 grit is relevant across different language learning contexts. Its relationships with engagement and emotion may nevertheless vary according to the educational and sociocultural conditions in which persistence is sustained.

L2 grit has also been associated with learners’ emotional experiences. Higher L2 grit has generally been linked to lower FLA and more adaptive language-learning outcomes (Sudina & Plonsky, 2021; Heydarnejad et al., 2022), suggesting that learners higher in L2 grit may be better able to sustain goal-directed involvement when experiencing anxiety. Yet continued persistence under demanding conditions is not necessarily uniformly adaptive. Experimental evidence indicates that highly gritty individuals may continue investing effort even when persistence becomes costly (Lucas et al., 2015), while more recent work suggests that persistence can become maladaptive when individuals fail to disengage from difficult or unattainable goals (Toyama, 2024). Together, these findings suggest that the role of grit may depend on the conditions under which persistence is sustained. This context dependence may be particularly relevant when learners experience anxiety in psychologically controlling learning environments, where the consequences of sustained persistence may be less straightforward. Existing studies have largely examined direct or indirect associations, leaving unclear whether L2 grit attenuates or intensifies the association between FLA and FLLE. This question is particularly relevant to the present model because variation in this association would also make the indirect association between PPCT and FLLE through FLA conditional on learners’ grit. Students’ attitudes toward English learning and teachers’ beliefs about language teaching may also shape how persistence and anxiety are experienced in the classroom, although these contextual factors were not assessed in the present study.

### 1.4. The Present Study

Building on the preceding literature, the present study examines the association between PPCT and FLLE among Chinese senior high school EFL learners, a context in which examination demands and classroom evaluation may make both teacher control and language-related anxiety particularly salient. Guided by SDT and CVT, we examine a moderated indirect association model in which FLA is examined as the variable involved in the indirect association between PPCT and FLLE, while L2 grit is considered a possible boundary condition of the association between FLA and FLLE.

To address these questions from complementary analytical perspectives, the present study combines structural equation modeling (SEM) with psychological network analysis (PNA). SEM tests the hypothesized relationships using an aggregate construct-level representation of FLLE while accounting for measurement error in the latent-variable framework. It examines the association between PPCT and FLLE, the indirect association through FLA, and the moderation of the association between FLA and FLLE by L2 grit. PNA provides a finer-grained perspective by mapping the conditional associations among PPCT, FLA, L2 grit, and the behavioral, cognitive, emotional, and agentic dimensions of FLLE after accounting for the remaining nodes in the network. This approach characterizes how the focal variables are selectively connected across engagement dimensions and identifies nodes occupying relatively central positions within the network. Together, the two analyses provide complementary construct-level and dimension-level accounts of the relationships under investigation.

Accordingly, we propose that (H1) PPCT is negatively associated with overall FLLE; (H2) PPCT is indirectly associated with FLLE through FLA, such that higher PPCT is associated with greater FLA, which is in turn associated with lower FLLE; and (H3) L2 grit moderates the association between FLA and FLLE. Given the limited evidence for dimension-specific patterns, we further examine how PPCT, FLA, and L2 grit are conditionally associated with the four dimensions of FLLE and which variables emerge as relatively central within the network.

## 2. Materials and Methods

### 2.1. Participants and Procedures

Participants were recruited from a public high school in Changsha, Hunan Province, China. The school was selected through convenience sampling based on accessibility and institutional willingness to participate. Within the school, eight Grade 10 classes were included as intact classroom groups, and students in these classes were invited to participate in the survey during scheduled self-study periods. Questionnaires were excluded if more than 20% of the questionnaire items were unanswered, an entire core study scale was left unanswered, more than 10 consecutive items received the same response, or an obvious wave-like or mechanically alternating response pattern was observed. After screening, 285 valid questionnaires were retained for analysis. Participant characteristics are summarized in Table 1.

Among the 285 retained questionnaires, 124 of the 13,110 responses across the 46 focal questionnaire items were missing (0.95%). These sporadic item-level missing responses were replaced using the participant’s mean on the remaining items of the corresponding scale, leaving no missing values in the analytic dataset.

All data were collected offline using paper-and-pencil questionnaires administered during self-study periods. Before participation, students received detailed instructions regarding the purpose of the study. The questionnaires were distributed and collected in class, and all participants completed the questionnaires anonymously in approximately 20 min. Students were informed that all data would be used only for academic research and kept strictly confidential. Ethical approval for this study was obtained from the Institutional Ethics Committee at the corresponding institution.

Following preliminary questionnaire development and subsequent refinement of the foreign language anxiety items, the final survey version used for data collection was completed in September 2024. Data were collected from October to November 2024. Prior to data collection, written informed consent was obtained from both the participating students and their legal guardians.

### 2.2. Measures

#### 2.2.1. Perceived Psychologically Controlling Teaching

Perceived psychologically controlling teaching was measured using the Psychologically Controlling Teaching Scale–Student Report developed by Soenens et al. (2012). The original items were contextualized for the EFL classroom by specifying the teacher as the students’ English teacher while retaining the substantive content of the original measure. The scale consists of 7 items that specifically assess students’ perceptions of psychologically controlling practices by their English teacher (e.g., “My English teacher is always trying to change me.”). Each item was rated on a 5-point Likert scale from 1 (strongly disagree) to 5 (strongly agree). Higher scores indicated greater perceived psychologically controlling teaching. In the present study, Cronbach’s alpha was 0.82.

#### 2.2.2. Foreign Language Learning Engagement

Foreign language learning engagement was assessed using the 22-item, four-dimensional engagement measure developed by Reeve and Tseng (2011), with the items contextualized for English classes following prior EFL applications of the measure (Wang et al., 2024). This scale has four dimensions: behavioral (5 items; e.g., “The first time my English teacher talks about a new topic, I listen very carefully”), cognitive (8 items; e.g., “Before I begin to study English, I think about what I want to get done”), emotional (4 items; e.g., “I enjoy learning new things in my English class”), and agentic (5 items; e.g., “I offer suggestions about how to make my English class better”). In the present study, each item was rated on a 5-point Likert scale. Higher scores indicated greater foreign language learning engagement. In the present study, Cronbach’s alpha was 0.92 for the overall scale and 0.93, 0.78, 0.70, and 0.88 for behavioral, cognitive, emotional, and agentic engagement, respectively.

#### 2.2.3. Foreign Language Anxiety

Foreign language anxiety was measured using the Chinese-adapted Short-Form Foreign Language Classroom Anxiety Scale (S-FLCAS; Botes et al., 2022; Dong & Huang, 2024). This scale consists of 8 self-report items (e.g., “I get nervous and confused when I am speaking in English class”), rated on a 5-point Likert scale from 1 (strongly disagree) to 5 (strongly agree). Two items (Items 4 and 5) were reverse scored (e.g., “I feel confident when I speak in FL class”) to mitigate response bias. Higher scores reflected greater levels of foreign language anxiety. In the present study, Cronbach’s alpha was 0.92. 

#### 2.2.4. L2 Grit

L2 grit was measured using the Chinese version of the L2 Grit Scale examined by E. Liu et al. (2022), originally developed by Teimouri et al. (2022). The scale consists of 9 items (e.g., “I work hard to learn English”), with items 2, 4, 7, and 8 reverse-scored (e.g., “My interest in learning English fluctuates over time”). Responses were recorded on a 5-point Likert scale, with higher scores indicating a greater level of L2 grit. In the present study, Cronbach’s alpha was 0.79. 

### 2.3. Data Analysis

Preliminary analyses were conducted in SPSS 27.0, including Harman’s single-factor test for potential common method bias, descriptive statistics, correlations, and examinations of univariate normality. For the descriptive analyses and PNA, all observed construct scores were calculated as mean item scores, with the four FLLE dimensions scored separately according to their respective items.

Confirmatory factor analysis (CFA) and structural equation modeling (SEM) were conducted in AMOS 28.0 using maximum likelihood estimation. The hypothesized four-factor measurement model was evaluated against alternative models, with convergent and discriminant validity assessed using composite reliability (CR), average variance extracted (AVE), and the Fornell–Larcker criterion. Model fit was evaluated using χ^2^/df, CFI, TLI, and RMSEA. PPCT was represented by two item parcels, whereas FLA and L2 grit were each represented by three parcels formed using item-to-construct balancing based on factor loadings (Little et al., 2013). For the primary SEM, the four FLLE subscale scores were used as indicators of an aggregate construct-level representation of FLLE to provide a parsimonious summary of overall engagement. This specification was used for the structural analysis and was not intended to establish a psychometrically confirmed general or higher-order FLLE factor. The dimensional structure of FLLE was examined separately at the item level and through dimension-specific sensitivity analyses. 

Structural analyses proceeded from an indirect-association model to a moderated indirect-association model. The latter tested whether L2 grit moderated the FLA–FLLE association and whether the indirect association between PPCT and FLLE through FLA varied across levels of L2 grit. The three FLA and three L2 grit parcels were mean-centered and paired to form three product indicators for the latent interaction term. Significant interactions were probed at low (−1 *SD*), mean, and high (+1 *SD*) levels of L2 grit. Indirect and conditional indirect associations were evaluated using 5000 bootstrap samples with bias-corrected 95% confidence intervals. A Monte Carlo sensitivity analysis was conducted using the parameter estimates of the final latent moderated indirect association model to evaluate the empirical power to detect a latent interaction of the observed magnitude at the available sample size. Additional sensitivity analyses examined the bifactor representation of L2 grit and the robustness of the moderation pattern to alternative grit operationalizations; full details are reported in Supplementary Section S5.

PNA was conducted in R 4.4.1. A seven-node network comprising PPCT, FLA, L2 grit, and the four FLLE dimensions was estimated using EBICglasso (γ = 0.50) in the qgraph package (Epskamp et al., 2012). The seven node scores were calculated after the item-level missing-data procedure described above, and no missing node scores remained before network estimation. The node scores were not standardized, and the network was estimated from Pearson correlations. Edges represented regularized partial correlations. Expected influence was used as the primary centrality index (Robinaugh et al., 2016). Edge accuracy and centrality stability were evaluated using 5000 nonparametric and case-dropping bootstrap samples in bootnet (Epskamp et al., 2018). Sensitivity analyses using γ = 0.25 and 0 were also conducted.

## 3. Results

### 3.1. Assessment of Potential Common Method Variance

Harman’s single-factor test (Podsakoff et al., 2003) was conducted as a diagnostic check for potential common method variance. An unrotated exploratory factor analysis extracted 10 factors with eigenvalues greater than 1, and the first factor accounted for 32.19% of the total variance. Thus, the analysis did not indicate the presence of a dominant single-factor structure. However, this diagnostic does not rule out common method variance. 

### 3.2. Descriptive Statistics and Correlation Analysis

Descriptive statistics and correlations are presented in Table 2. PPCT was negatively correlated with FLLE (*r* = −0.31, *p* < 0.01) and L2 grit (*r* = −0.26, *p* < 0.01), but positively correlated with FLA (*r* = 0.30, *p* < 0.01). FLLE was positively correlated with L2 grit (*r* = 0.60, *p* < 0.01) and negatively correlated with FLA (*r* = −0.45, *p* < 0.01). FLA was also negatively correlated with L2 grit (*r* = −0.49, *p* < 0.01). Gender was negatively correlated with PPCT (*r* = −0.27, *p* < 0.01) and positively correlated with FLA (*r* = 0.12, *p* < 0.05), whereas age was unrelated to the focal variables. Across the four continuous focal variables, skewness ranged from −0.29 to 1.17 and kurtosis ranged from 0.44 to 2.54, indicating no severe departures from univariate normality. Overall, higher PPCT was associated with greater FLA and lower FLLE and L2 grit, whereas higher L2 grit was associated with greater FLLE and lower FLA. These bivariate patterns were examined further in the subsequent SEM analyses.

### 3.3. Measurement Model and Construct Validity

A series of confirmatory factor analyses was conducted to examine the measurement structure of the four focal constructs. The hypothesized four-factor model, in which PPCT, FLA, L2 grit, and FLLE were modeled as separate but correlated latent factors, showed an acceptable fit to the data, χ^2^(48) = 106.40, *p* < 0.001, χ^2^/df = 2.22, CFI = 0.969, TLI = 0.957, and RMSEA = 0.065, 90% CI [0.049, 0.082]. Standardized factor loadings ranged from 0.639 to 0.889 (all *p* < 0.001). Composite reliability values ranged from 0.835 to 0.875 and AVE values from 0.638 to 0.718, supporting adequate reliability and convergent validity at the aggregated indicator level used in the primary SEM. The square root of the AVE for each construct (from 0.799 to 0.847) exceeded all absolute interconstruct correlations. Construct-specific reliability, convergent-validity, and discriminant-validity statistics are reported in Supplementary Table S1.

The hypothesized model was compared with two alternative three-factor models (combining L2 grit with FLLE and PPCT with FLA, respectively) and a one-factor model. All alternative models showed poorer fit than the hypothesized four-factor model (Table 3). Taken together, these results supported the empirical separability of PPCT, FLA, L2 grit, and the aggregate FLLE representation at the level used in the primary SEM. This parcel-level fit does not establish that FLLE is unidimensional at the item level or that its four dimensions form a psychometrically validated higher-order factor. Item-level analyses instead favored a correlated four-factor representation over a one-factor model, although absolute fit remained limited, and the higher-order FLLE model yielded an improper solution. Accordingly, the FLLE factor in the primary SEM is interpreted as a pragmatic aggregate representation rather than a confirmed general FLLE construct. Full item-level analyses of L2 grit and FLLE are reported in Supplementary Sections S5 and S6, respectively.

### 3.4. Structural Equation Modeling

Using the parcel-level construct representation specified for the primary SEM, the structural relationships were examined in two stages. First, an indirect association model was estimated to examine the direct association between PPCT and FLLE, as well as their indirect association through foreign language anxiety (FLA). Second, a moderated indirect association model was estimated to test whether L2 grit moderated the association between FLA and FLLE and, consequently, the indirect association between PPCT and FLLE through FLA.

#### 3.4.1. Indirect Association Through Foreign Language Anxiety

The indirect association model demonstrated acceptable fit: χ^2^(24) = 63.97, *p* < 0.001, χ^2^/df = 2.666, CFI = 0.970, TLI = 0.955, RMSEA = 0.077, 90% CI [0.054, 0.100]. PPCT was negatively associated with FLLE (*β* = −0.242, *p* < 0.001) and positively associated with FLA (*β* = 0.314, *p* < 0.001). FLA was also negatively associated with FLLE (*β* = −0.421, *p* < 0.001). Bootstrap tests confirmed that the indirect association between PPCT and FLLE through FLA was significant (*β* = −0.132, *p* < 0.001, 95% CI [−0.211, −0.077]), accounting for approximately 35.3% of the total association (total association = −0.374). Thus, H1 and H2 were supported. 

#### 3.4.2. Moderation by L2 Grit

**Figure 1:**
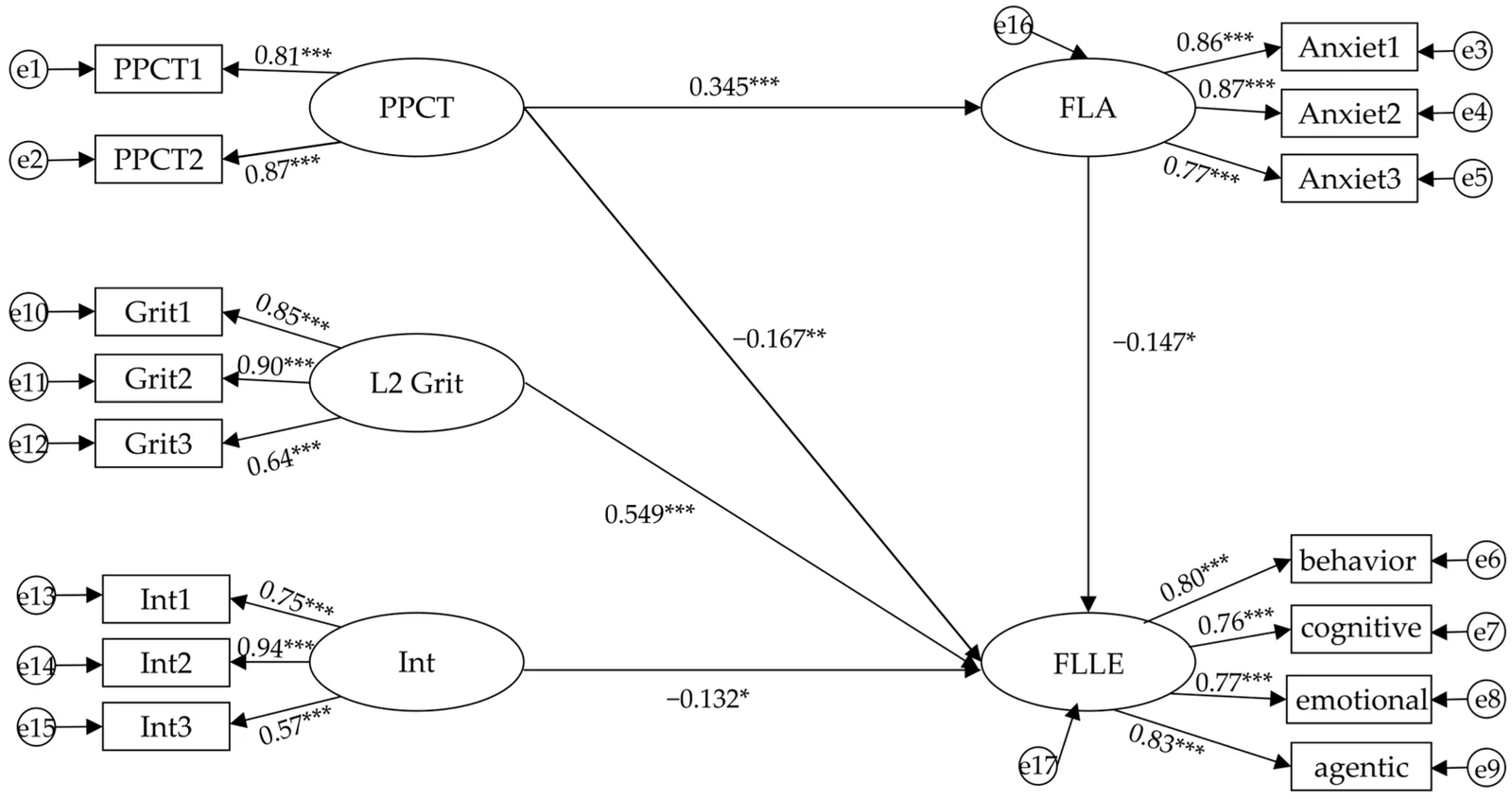
Structural equation model. Note. *N* = 285, * *p* < 0.05, ** *p* < 0.01, *** *p* < 0.001. The path estimates are standardized. PPCT = Perceived psychologically controlling teaching; FLA = Foreign language anxiety; FLLE = Foreign language learning engagement; Int = latent FLA × L2 grit interaction. FLLE represents the aggregate construct-level specification used in the primary SEM and should not be interpreted as a psychometrically confirmed higher-order factor.

Figure 1 displays the results of the moderated indirect association model. Its fit was somewhat weaker than that of the measurement and indirect association models but remained acceptable: χ^2^(82) = 229.85, *p* < 0.001, χ^2^/df = 2.803, CFI = 0.933, TLI = 0.914, RMSEA = 0.080, 90% CI [0.068, 0.092]. PPCT was positively associated with FLA (*β* = 0.345, *p* < 0.001) and negatively associated with FLLE (*β* = −0.167, *p* = 0.009). FLA was negatively associated with FLLE (*β* = −0.147, *p* = 0.011), whereas L2 grit was positively associated with FLLE (*β* = 0.549, *p* < 0.001). The interaction between FLA and L2 grit was significantly associated with FLLE (*β* = −0.132, *p* = 0.016, 95% CI [−0.248, −0.013]). 

**Figure 2:**
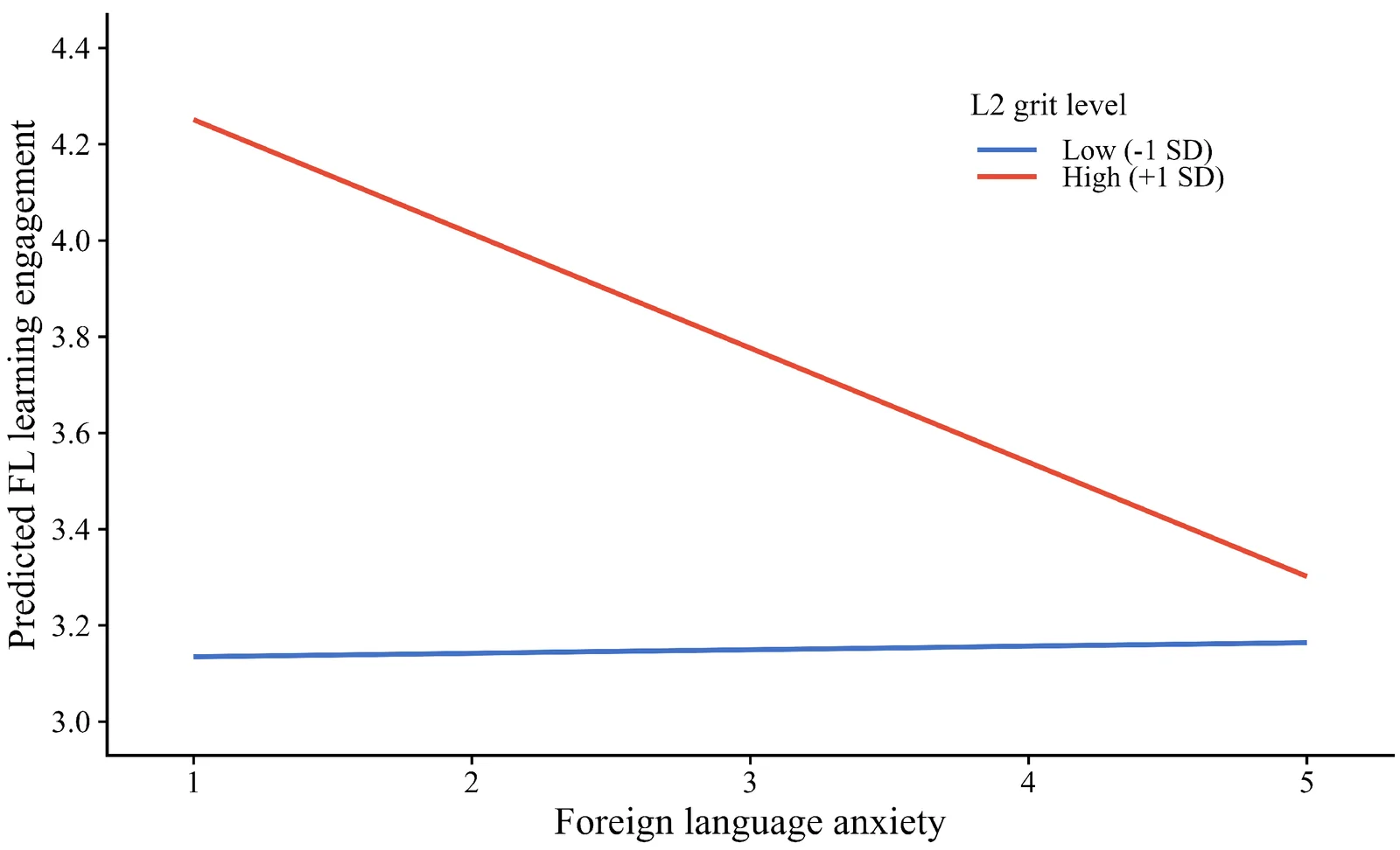
Moderating effects of L2 grit on the association between FLA and FLLE.

As illustrated in Figure 2, simple-slope analysis showed that FLA was negatively associated with FLLE at high levels of L2 grit (*b* = −0.237, *p* = 0.004, 95% CI [−0.409, −0.077]). In contrast, the corresponding associations at low (*b* = 0.007, *p* = 0.948, 95% CI [−0.158, 0.203]) and mean (*b* = −0.115, *p* = 0.056, 95% CI [−0.224, 0.002]) levels of L2 grit were not statistically significant. As shown in Table 4, the conditional indirect association between PPCT and FLLE through FLA was nonsignificant at low levels of L2 grit (*b* = 0.003, *p* = 0.940, 95% CI [−0.067, 0.079]), but significant at mean (*b* = −0.045, *p* = 0.044, 95% CI [−0.105, −0.002]) and high levels (*b* = −0.094, *p* = 0.003, 95% CI [−0.184, −0.036]). More importantly, the index of moderated mediation was significant (index = −0.071, *p* = 0.028, 95% CI [−0.168, −0.009]), indicating that the negative indirect association became stronger as L2 grit increased. These results supported H3 by demonstrating moderation, although the observed pattern did not indicate a buffering role of L2 grit. 

The Monte Carlo sensitivity analysis indicated an empirical power of approximately 0.64 for detecting an interaction of the observed magnitude at *N* = 285. A gender-adjusted sensitivity model showed that the primary moderation effect remained comparable in magnitude. Gender was positively associated with FLA (*β* = 0.260, *p* < 0.001) but not with FLLE (*β* = −0.035, *p* = 0.544), and the interaction between FLA and L2 grit remained significant and nearly identical in magnitude (*β* = −0.132, *p* = 0.015). Full sensitivity results are reported in the Supplementary Materials. The moderation pattern was also reproduced across alternative operationalizations of L2 grit, including perseverance of effort, consistency of interest, and an eight-item general-grit score excluding Item 2 (Supplementary Section S5).

#### 3.4.3. Dimension-Specific Sensitivity Analyses

Given the multidimensional structure of FLLE indicated by the item-level analyses, exploratory sensitivity analyses examined the moderated indirect association separately for behavioral, cognitive, emotional, and agentic engagement. Before adjustment for multiple comparisons, the FLA × L2 grit interaction was nominally significant for cognitive (*p* = 0.028), emotional (*p* = 0.006), and agentic engagement (*p* = 0.039), but not for behavioral engagement (*p* = 0.080). After Holm correction for the four interaction tests, only the interaction for emotional engagement remained statistically significant (Holm-adjusted *p* = 0.024; all other Holm-adjusted *p* values = 0.084). The bootstrap confidence interval for the index of moderated indirect association excluded zero for emotional and agentic engagement but included zero for behavioral and cognitive engagement. Thus, the aggregate conditional pattern was not reproduced uniformly across the four engagement dimensions. Full estimates are reported in Supplementary Table S12.

### 3.5. Psychological Network Analysis

**Figure 3:**
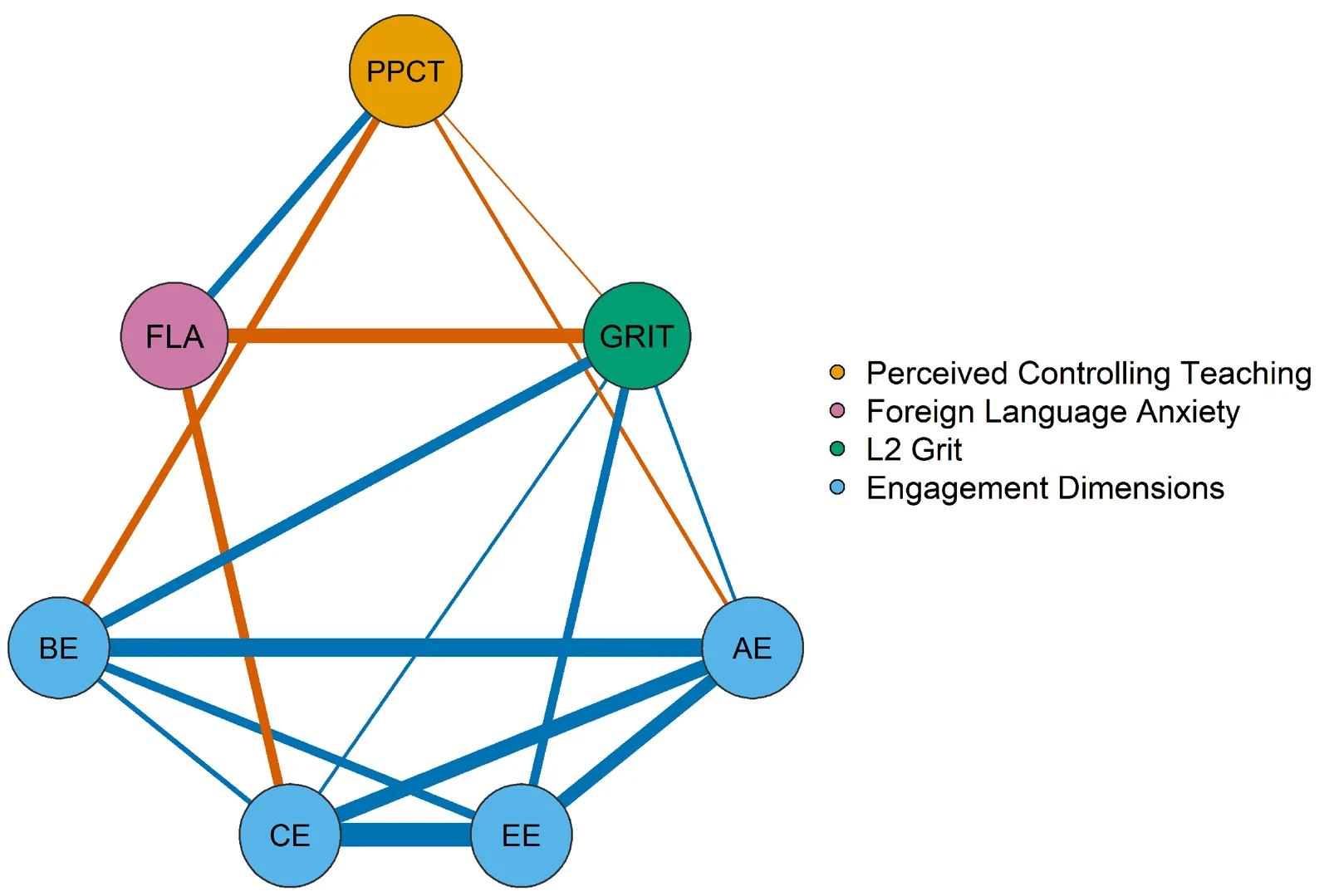
Psychological network structure. Edges represent regularized partial correlations, with blue and red edges indicating positive and negative associations, respectively. Thicker edges indicate larger absolute weights. PPCT = perceived psychologically controlling teaching; FLA = foreign language anxiety; GRIT = L2 grit; BE = behavioral engagement; CE = cognitive engagement; EE = emotional engagement; AE = agentic engagement.

Whereas the SEM analyses examined the hypothesized relationships using the aggregate construct-level representation of FLLE, the PNA was used to examine whether these associations showed more selective patterns across the four dimensions of FLLE. The seven-node network retained 16 of 21 possible edges, including 11 positive and five negative edges (Figure 3). The strongest connections were between cognitive and emotional engagement (CE–EE, weight = 0.459), behavioral and agentic engagement (BE–AE, weight = 0.347), and cognitive and agentic engagement (CE–AE, weight = 0.292). FLA was negatively connected with L2 grit (weight = −0.273) and cognitive engagement (weight = −0.170), whereas L2 grit was positively connected with behavioral (weight = 0.207) and emotional engagement (weight = 0.159). PPCT was positively connected with FLA (weight = 0.156) and negatively connected with behavioral engagement (weight = −0.137), with a weaker negative edge to agentic engagement (weight = −0.049). No edges were retained between PPCT and cognitive or emotional engagement, or between FLA and behavioral, emotional, or agentic engagement.

**Figure 4:**
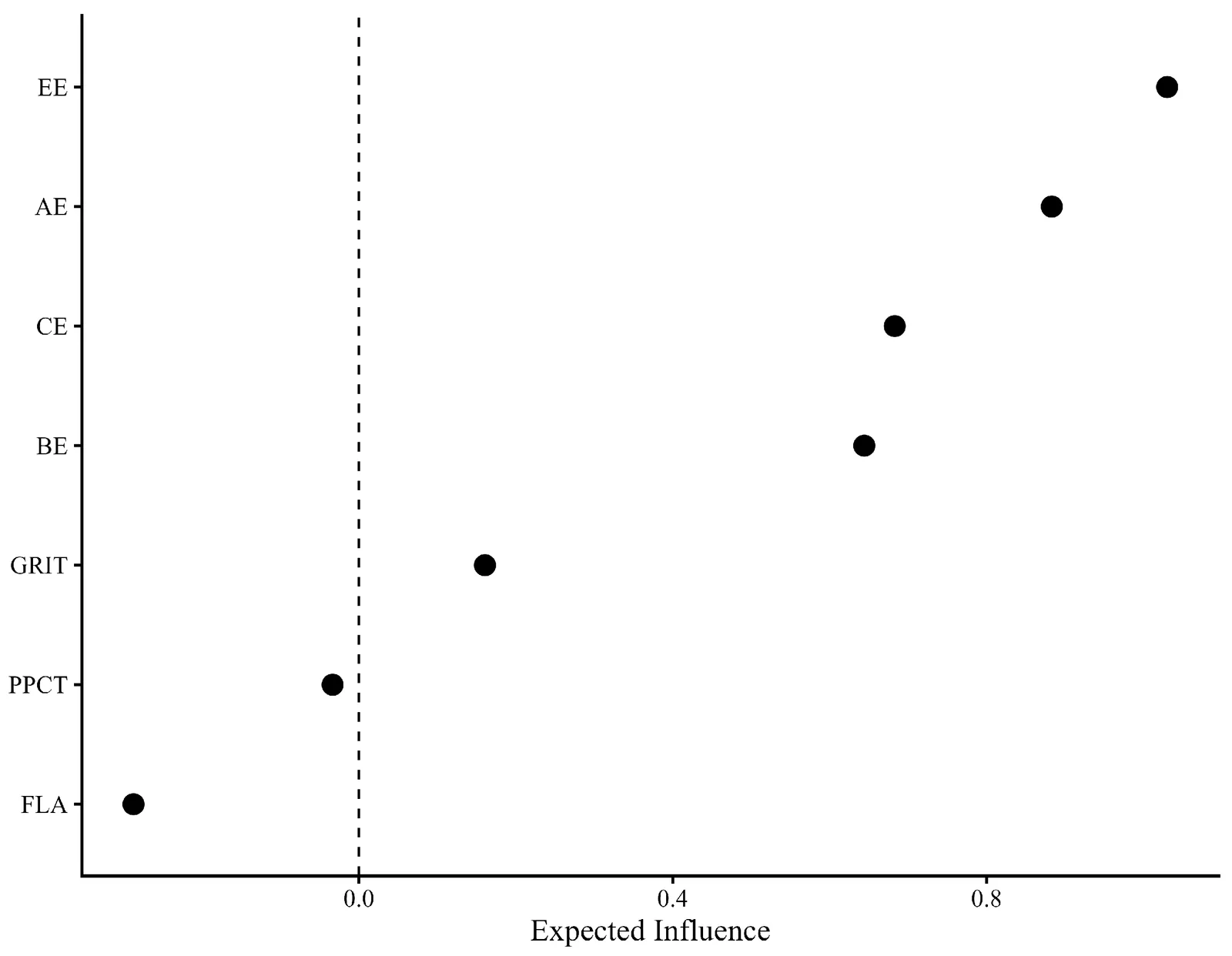
Expected influence of the seven nodes in the psychological network. Higher positive values indicate a stronger net pattern of positive connections with other nodes, whereas negative values indicate a stronger net pattern of negative connections. The dashed vertical line represents zero. PPCT = perceived psychologically controlling teaching; FLA = foreign language anxiety; GRIT = L2 grit; BE = behavioral engagement; CE = cognitive engagement; EE = emotional engagement; AE = agentic engagement.

Emotional engagement showed the highest expected influence (EI = 1.030), followed by agentic engagement (EI = 0.883), cognitive engagement (EI = 0.683), and behavioral engagement (EI = 0.644). L2 grit showed a modest positive expected influence (EI = 0.161), PPCT showed an expected influence close to zero (EI = −0.033), and FLA showed the most negative expected influence (EI = −0.287; Figure 4).

Nonparametric bootstrap results indicated that the estimates of the stronger edges were relatively stable, although overlapping and comparatively wide confidence intervals for several edges precluded fine-grained comparisons between edges of similar magnitude (Figures S1 and S2). Case-dropping bootstrap analysis indicated high stability for expected influence (CS = 0.751; Figure S3). Sensitivity analyses yielded an identical network at γ = 0.25; when γ was set to 0, only one additional weak PPCT–EE edge was retained (weight = 0.031; Table S8), indicating that the principal network structure was robust across tuning parameters.

## 4. Discussion

The present study identified three complementary patterns in how PPCT, FLA, L2 grit, and engagement were associated among Chinese senior high school EFL learners. First, at the aggregate construct level, PPCT showed both a direct negative association with FLLE and an indirect association through FLA. Second, L2 grit moderated the aggregate FLA–FLLE association, but the corresponding conditional pattern was heterogeneous across engagement dimensions rather than uniformly reproduced across behavioral, cognitive, emotional, and agentic engagement. The most consistent dimension-specific evidence was observed for emotional engagement. Third, PNA further showed that PPCT, FLA, and L2 grit occupied selective positions within the network of engagement dimensions. Together, these findings indicate that the aggregate SEM captures a broad construct-level pattern, whereas the dimension-specific analyses reveal substantial differentiation within FLLE.

### 4.1. The Negative Association Between Psychologically Controlling Teaching and FLLE

The negative direct association between PPCT and FLLE supported H1 and remained significant when FLA was included in the model. This finding is consistent with previous evidence that controlling teaching is associated with lower student engagement across educational contexts, including recent longitudinal evidence from Chinese secondary schools and findings from EFL classrooms (Jiang & Zhang, 2021; Dammert et al., 2025; Yue et al., 2026). The present study extends this pattern to aggregate FLLE among Chinese senior high school EFL learners.

SDT provides one theoretical account of this association by proposing that psychological control may weaken engagement through the frustration of students’ basic psychological needs and a shift toward more controlled forms of regulation (Ryan & Deci, 2020). Longitudinal evidence is consistent with this process, showing that controlling teaching is associated with greater need frustration and, in turn, poorer-quality motivation and avoidance-oriented responses (Bartholomew et al., 2018). More recent work similarly found that teacher control was linked to external regulation through need frustration and was negatively associated with behavioral and agentic engagement (Dammert et al., 2025).

This theoretical pathway may be particularly consequential in language learning, where sustained engagement depends on continued interaction, effort, and willingness to use the target language despite uncertainty and possible errors. EFL research supports the importance of this motivational quality of participation, with controlling teaching linked to lower agentic engagement and autonomy-supportive teaching associated with stronger classroom engagement (Jiang & Zhang, 2021; Wang et al., 2024). In examination-oriented senior high school classrooms, external demands may be sufficient to keep students on task, but such participation does not necessarily involve the initiative or personal investment required for sustained language learning. The negative association observed here may therefore reflect not simply whether students participate, but how actively and willingly they engage with English learning.

### 4.2. Foreign Language Anxiety as an Indirect Pathway

The significant indirect association through FLA supported H2. Students who perceived greater psychological control from their English teachers also reported higher FLA, which was in turn associated with lower engagement at the aggregate construct level. This finding is consistent with the hypothesized indirect association involving FLA and fits the broader pattern reported in previous research, in which controlling classroom experiences are associated with anxiety-related responses and FLA is generally linked to poorer engagement in language learning (Assor et al., 2005; Qian & Huang, 2026; X. Li et al., 2024).

The positive association between PPCT and FLA is particularly meaningful in a language classroom, where students regularly display their language competence through speaking, answering questions, and other evaluative classroom activities. This pattern is consistent with the theoretical account proposed earlier based on SDT and CVT, whereby psychological pressure can undermine students’ autonomy, competence, and perceived control over learning and performance (Ryan & Deci, 2020; Pekrun, 2024). Psychological control can further raise the interpersonal stakes of mistakes or poor performance by tying them more closely to teacher approval, disappointment, or students’ sense of competence. This interpretation is supported by evidence linking controlling teaching to fear of negative evaluation and related anxiety vulnerabilities, as well as findings that lower perceived control is associated with greater anxiety among Chinese high school EFL learners (Salazar-Ayala et al., 2021; Qian & Huang, 2026).

The negative association between FLA and FLLE further indicates that anxiety is closely tied to how fully students engage in English learning. Worry about mistakes, evaluation, and performance can compete for attention and make active participation more effortful, particularly when students must continue using the target language despite uncertainty. This interpretation accords with evidence showing lower engagement among learners experiencing greater FLA (X. Li et al., 2024; Chen & Guo, 2026). In the present model, FLA therefore represents an important emotional correlate within the indirect association between students’ perceptions of psychological control and lower engagement.

### 4.3. The Conditional Role of L2 Grit

In the primary aggregate model, L2 grit significantly moderated the association between FLA and FLLE, supporting H3. Although L2 grit was positively associated with FLLE overall, its moderating role did not reflect the expected protective pattern. Instead, the relationship became more negative as grit increased, with little association at low grit and a clear negative association at high grit. The conditional indirect association between PPCT and FLLE through FLA showed the same tendency, becoming more negative as grit increased and reaching significance at average and high levels. This finding qualifies the predominantly positive view of L2 grit, which has generally been associated with greater engagement and willingness to communicate (Jin, 2024; G. Li, 2024). The psychometric interpretation of L2 grit nevertheless requires caution. The bifactor representation did not yield a stable admissible solution in the present sample, whereas the moderation pattern was reproduced across alternative grit operationalizations. Thus, the present evidence supports the robustness of the moderation finding more strongly than it supports a psychometrically homogeneous general L2 grit construct.

The dimension-specific sensitivity analyses further qualify the aggregate moderation pattern by indicating that the interplay between FLA and L2 grit is not expressed uniformly across forms of engagement. The comparatively stronger evidence for emotional engagement suggests that the conditional role of grit may be more closely tied to learners’ affective involvement than to all aspects of classroom engagement equally. Evidence for the remaining dimensions was less consistent, indicating that the aggregate FLLE result may partly reflect the combined contribution of qualitatively different engagement processes rather than a single common mechanism. This heterogeneity is consistent with the multidimensional conceptualization of engagement and cautions against interpreting the construct-level moderation as applying equally to behavioral, cognitive, emotional, and agentic engagement.

One plausible account concerns the persistence inherent in grit. Grittier individuals tend to continue pursuing difficult goals even when persistence becomes costly (Lucas et al., 2015; Toyama, 2024). When anxiety remains high, however, continuing to perform may require additional compensatory effort. Attentional control theory proposes that anxiety reduces processing efficiency, so maintaining performance often requires greater effort and cognitive resources (Eysenck et al., 2007). Evidence from secondary-school examinations further suggests that additional effort does not always offset the effects of worry when cognitive demands are high (Putwain & Symes, 2018). Persistent learners may therefore remain engaged in goal pursuit while continuing to bear the cognitive costs associated with anxiety.

This account may be particularly relevant in evaluative EFL classrooms, where classroom conditions and perceived control are closely related to fluctuations in anxiety (Qian & Huang, 2026). Under psychologically controlling teaching, higher-grit learners may continue pursuing their language-learning goals while evaluation, possible errors, and uncertainty remain salient. Anxiety may therefore continue to interfere with the resources needed for effective engagement even as effort is sustained. Viewed from the other side of the interaction, the engagement advantage associated with higher grit becomes less pronounced as FLA increases. Because persistence, compensatory effort, and cognitive resource expenditure were not measured directly, this interpretation remains tentative. Grit may help learners persist, but persistence does not necessarily protect engagement when anxiety remains high.

### 4.4. Conditional Associations Across Engagement Dimensions Revealed by PNA 

The PNA further differentiated the construct-level associations identified by SEM. Whereas SEM showed a negative association between PPCT and aggregate FLLE, the network revealed that, after controlling for all other nodes, PPCT retained its clearest negative connection with behavioral engagement and a weaker connection with agentic engagement, while no edges were retained with cognitive or emotional engagement. Thus, the negative association between PPCT and engagement was not evenly distributed across dimensions and was most clearly evident for behavioral engagement. This pattern aligns with previous evidence linking controlling teaching to lower agentic engagement in Chinese EFL classrooms (Jiang & Zhang, 2021) and lower behavioral engagement among Chinese secondary-school students (Yue et al., 2026). The concentration of these conditional associations in behavioral and agentic engagement may also reflect features of the local instructional context. One possible contextual interpretation is that, in Chinese EFL classrooms, examination demands and teacher-directed classroom structures may sustain observable participation without necessarily producing equivalent levels of deeper cognitive, emotional, or agentic involvement (Wang & Liu, 2022; Zhang & Hu, 2025).

A similarly selective pattern emerged for FLA and L2 grit. Although FLA was negatively associated with aggregate FLLE in the SEM, it retained a negative network connection only with cognitive engagement. Because cognitive engagement relies heavily on sustained attention, strategic effort, and deeper processing, this finding is consistent with evidence that anxiety disrupts attentional control and reduces processing efficiency (Eysenck et al., 2007). L2 grit, in contrast, remained positively connected with behavioral and emotional engagement. Previous EFL research has linked L2 grit to stronger classroom engagement overall (Jin, 2024), while more recent evidence has shown positive associations with behavioral, cognitive, and emotional engagement specifically (Derakhshan & Datu, 2025). However, in the present network only its connections with behavioral and emotional engagement remained after the other nodes were controlled. The positive connection between PPCT and FLA also mirrored the corresponding SEM association, while FLA and L2 grit remained negatively connected within the broader network.

The strongest connections occurred among the engagement dimensions themselves, particularly between cognitive and emotional engagement, behavioral and agentic engagement, and cognitive and agentic engagement. Emotional engagement also showed the highest estimated expected influence. Together, these findings suggest that the four dimensions of FLLE were closely interconnected while retaining differentiated relationships with PPCT, FLA, and L2 grit. This pattern is consistent with meta-analytic evidence that different forms of engagement serve partly specialized functions in students’ learning experiences (Reeve et al., 2025). The relatively high estimated expected influence of emotional engagement indicates that it had the strongest net pattern of signed connections among the seven nodes in this network. This centrality pattern is descriptive and should not be interpreted as evidence that intervening on emotional engagement would necessarily produce the largest changes elsewhere in the network. Overall, the PNA refines the SEM findings by revealing a more selective dimension-level organization beneath the broader construct-level associations with FLLE. This dimension-level differentiation is also consistent with the sensitivity analyses of the moderated indirect model, which showed that the aggregate conditional pattern was not reproduced uniformly across all four engagement dimensions.

### 4.5. Theoretical and Practical Implications

The findings have several theoretical implications. First, they are consistent with an integrated account drawing on SDT and CVT by identifying a significant indirect association involving FLA. This connects SDT’s emphasis on controlling interpersonal environments with CVT’s explanation of anxiety under conditions of reduced perceived control, extending their joint relevance to senior high school EFL engagement. Second, the moderation results qualify the predominantly positive conceptualization of L2 grit. Although grit was positively associated with engagement overall, it did not function as a uniform protective resource against anxiety, suggesting that its role depends partly on the emotional conditions under which persistence is sustained. Third, the dimension-specific sensitivity analyses and PNA jointly extend the multidimensional view of FLLE by showing that broad construct-level associations can conceal heterogeneous dimension-level patterns. The conditional role of L2 grit was not expressed uniformly across behavioral, cognitive, emotional, and agentic engagement, while the network analysis further showed that PPCT, FLA, and L2 grit occupied differentiated positions in relation to these dimensions. This convergence across analytical perspectives reinforces the view that the engagement dimensions are interconnected but not interchangeable. These theoretical implications should be interpreted within the present Chinese senior high school EFL context, as the relationships among psychological control, anxiety, grit, and engagement may take different forms across language education settings.

For classroom practice, the findings highlight the importance of reducing psychological control. Teachers should avoid guilt induction, shaming, conditional approval, or expressions of disappointment that target students’ self-worth. Moreover, classroom evaluation should make errors less threatening through constructive feedback and lower-risk opportunities to participate. The strong interconnections involving emotional engagement in the estimated network also underscore the relevance of students’ emotional experience in the classroom, although these network associations do not establish intervention effects. Finally, the findings on L2 grit call for a balanced view of persistence, as perseverance should be encouraged while teachers remain attentive to whether sustained effort is accompanied by excessive anxiety.

### 4.6. Limitations and Future Directions

Several limitations indicate directions for future research. The cross-sectional design does not establish temporal ordering, so longitudinal or intensive repeated-measures studies are needed to examine how PPCT, FLA, grit, and engagement develop over time. Although SDT and CVT provided theoretical explanations for the observed associations, basic psychological need frustration and control and value appraisals were not directly measured. The proposed mechanisms should therefore be tested explicitly in future research. The sample came from one Chinese public senior high school and relied primarily on student self-reports. Participants were also nested within eight classes, but consistent classroom and teacher identifiers were not available for analysis, so possible nonindependence at the classroom or teacher level could not be evaluated. Replication across schools and regions, combined with classroom observation and teacher reports, would strengthen generalizability and broaden the sources of evidence. Future research could also examine students’ attitudes toward English learning and teachers’ beliefs about language teaching, as these contextual factors may shape how psychological control, anxiety, grit, and engagement are experienced in EFL classrooms. Longitudinal network models could further determine whether the dimension-specific conditional associations identified here remain stable or change over time. The available sample provided moderate rather than high power for detecting the latent interaction, so the moderation finding warrants replication in larger samples. Although Harman’s single-factor analysis did not indicate a dominant single-factor structure, all focal variables were assessed through student self-report questionnaires administered on the same occasion, so common-source measurement may still have contributed to some of the observed associations.

## 5. Conclusions

This study combined SEM and PNA to examine how perceived psychologically controlling teaching is associated with foreign language learning engagement among Chinese senior high school EFL learners. At the aggregate construct level, PPCT was associated with lower FLLE both directly and indirectly through greater FLA, and the negative association between FLA and FLLE became stronger at higher levels of L2 grit. Dimension-specific analyses, however, showed that this conditional pattern was heterogeneous rather than uniform across behavioral, cognitive, emotional, and agentic engagement, with the most consistent evidence observed for emotional engagement. The network findings further showed that PPCT, FLA, and L2 grit were selectively connected with different engagement dimensions, with emotional engagement showing the highest expected influence. Overall, the findings support interpreting the primary SEM as an aggregate construct-level summary while recognizing that the internal organization of engagement is multidimensional and that the observed conditional associations vary across engagement dimensions.

## Figures and Tables

**Table 1:** Participant characteristics.

Characteristic	Description
Grade	Grade 10
Gender	Male, 160 (56.1%); Female, 125 (43.9%)
Age	*M* = 15.18, *SD* = 0.99; range = 14–17 years
Ethnicity	Han Chinese
Location	Changsha, Hunan Province, China

Note: *N* = 285.

**Table 2:** Descriptive statistics and Pearson correlations among all variables.

Variables	*M*	*SD*	1	2	3	4	5	6
1. Gender	0.44	0.50						
2. Age	15.18	0.99	−0.02					
3. PPCT	1.85	0.67	−0.27 **	−0.11				
4. FLLE	3.47	0.71	−0.02	0.05	−0.31 **			
5. FLA	2.94	0.81	0.12 *	−0.08	0.30 **	−0.45 **		
6. L2 grit	3.20	0.61	−0.07	0.04	−0.26 **	0.60 **	−0.49 **	
Skewness					1.17	0.93	−0.29	0.33
Kurtosis					2.54	1.51	0.93	0.44

Note: * *p* < 0.05, ** *p* < 0.01; Gender (“0” = male, “1” = female) as dummy variable; PPCT = perceived psychologically controlling teaching, FLA = Foreign language anxiety, FLLE = Foreign language learning engagement.

**Table 3:** Fit Indices for the hypothesized and alternative measurement models.

Model	χ^2^	df	χ^2^/df	CFI	TLI	RMSEA [90% CI]
Hypothesized four-factor model	106.40	48	2.22	0.969	0.957	0.065 [0.049, 0.082]
Three-factor model A: L2 grit and FLLE collapsed	285.70	51	5.6	0.874	0.838	0.127 [0.113, 0.142]
Three-factor model B: PPCT and FLA collapsed	302.68	51	5.94	0.865	0.826	0.132 [0.118, 0.146]
One-factor model: all constructs collapsed	743.58	54	13.77	0.631	0.549	0.212 [0.199, 0.226]

**Table 4:** Conditional Indirect Associations.

Model	L2 Grit Level	Estimate	Boot SE	95 % Bootstrap CI	*p*
Conditional indirect association	Low (−1 *SD*)	0.003	0.037	[−0.067, 0.079]	0.940
	Mean	−0.045	0.026	[−0.105, −0.002]	0.044
	High (+1 *SD*)	−0.094	0.039	[−0.184, −0.036]	0.003
Index of moderated mediation	—	−0.071	0.041	[−0.168, −0.009]	0.028

Note: Boot SE = bootstrap standard error. Bias-corrected 95% bootstrap confidence intervals were based on 5000 resamples.

## Data Availability

The data that support the findings of this study are not publicly available due to privacy and ethical restrictions involving human participants, including minor students, but are available from the corresponding author upon reasonable request.

## Supplementary Materials

The following supporting information can be downloaded at: https://www.mdpi.com/article/10.3390/bs16091662/s1.
