# Supplementary material for: Perceived Psychologically Controlling Teaching and Foreign Language Learning Engagement: Indirect Associations Through Foreign Language Anxiety and the Moderating Role of L2 Grit
Source: Behav Sci (Basel). 2026 Sep 16;16(9):1662. doi: 10.3390/bs16091662 (PMC13603977; doi:10.3390/bs16091662)
Supplement: Supplementary file 1 [file behavsci-16-01662-s001.zip › behavsci-4505069-supplementary.pdf]

Supplementary Materials

Perceived Psychologically Controlling Teaching and Foreign Language Learning Engagement

Contents

Section S1. Reliability, convergent validity, and discriminant validity

Table S1. Reliability, convergent validity, and discriminant validity of the focal constructs

Section S2. Structural parameter estimates

Table S2. Structural parameter estimates for the primary moderated indirect-association model

Section S3. Gender-adjusted sensitivity analysis

Table S3. Gender-adjusted latent moderated indirect-association model

Section S4. Psychological network analysis

Table S4. Zero-order Pearson correlations among the seven network nodes

Table S5. Regularized partial-correlation edge weights in the primary seven-node network (γ = .50)

Table S6. Expected influence scores for the seven network nodes

Table S7. Correlation-stability coefficients for centrality indices

Table S8. Sensitivity of the estimated network to the EBIC tuning parameter

Figure S1. Accuracy of the edge-weight estimates

Figure S2. Bootstrapped edge-weight difference test

Figure S3. Case-dropping bootstrap stability of expected influence

Supplementary Note S1. Key R syntax for psychological network analysis

Section S5. Item-level measurement and sensitivity analyses of L2 grit

Table S9. Item-level CFA results for L2 grit

Table S10. Gender-adjusted dimension-specific moderated indirect-association models

Table S10A. Selected results from the L2 grit bifactor sensitivity analyses

Table S10B. Moderated indirect-association sensitivity analysis using the eight-item grit score

Section S6. Item-level confirmatory factor analyses of foreign language learning engagement

Table S11. Comparison of alternative item-level measurement models for FLLE

Table S12. Dimension-specific sensitivity analyses of the moderated indirect association

Section S1. Reliability, convergent validity, and discriminant validity

Table S1. Reliability, convergent validity, and discriminant validity of the focal constructs

Construct	Standardized loading range	CR	AVE	PPCT	FLA	L2 grit	FLLE
PPCT	.811–.882	.835	.718	**.847**			
FLA	.767–.868	.872	.695	.314	**.834**		
L2 grit	.639–.889	.842	.645	−.296	−.565	**.803**	
FLLE	.770–.843	.875	.638	−.376	−.496	.683	**.799**

Note. CR = composite reliability; AVE = average variance extracted; PPCT = perceived psychologically controlling teaching; FLA = foreign language anxiety; FLLE = foreign language learning engagement. Bold diagonal values represent the square roots of AVE; off-diagonal values represent latent-factor correlations.

Section S2. Structural parameter estimates

Table S2. Structural parameter estimates for the primary moderated indirect-association model

**Structural path**	**b**	**SE**	**β**	**p**	**BC bootstrap 95% CI for β**
PPCT → FLA	.395	.080	.345	< .001	[.215, .466]
FLA → FLLE	−.115	.045	−.147	.011	[−.299, .004]
PPCT → FLLE	−.150	.057	−.167	.009	[−.307, .000]
L2 grit → FLLE	.472	.057	.549	< .001	[.383, .702]
FLA × L2 grit → FLLE	−.180	.075	−.132	.016	[−.248, −.013]

Note. N = 285. b = unstandardized coefficient; β = standardized coefficient. p values are based on maximum-likelihood estimates. Confidence intervals are bias-corrected bootstrap 95% intervals based on 5,000 usable bootstrap samples.

Section S3. Gender-adjusted sensitivity analysis

To examine whether the principal structural and moderation findings were robust to gender differences, the primary latent moderated indirect-association model was re-estimated with gender included as a covariate. Gender was specified as a predictor of both FLA and FLLE and was allowed to covary with PPCT, L2 grit, and the latent interaction factor. The model showed acceptable fit, $\chi^2(92) = 237.85$, $\chi^2/df = 2.59$, CFI = .935, TLI = .915, and RMSEA = .075, 90% CI [.063, .086]. Gender was positively associated with FLA but was not significantly associated with FLLE. Importantly, the FLA × L2 grit interaction remained negative and statistically significant and was essentially unchanged in magnitude relative to the primary model.

Table S3. Gender-adjusted latent moderated indirect-association model

**Structural path**	**b**	**SE**	**β**	**p**
PPCT → FLA	.480	.082	.429	< .001
Gender → FLA	.393	.096	.260	< .001
FLA → FLLE	−.105	.047	−.134	.026
PPCT → FLLE	−.164	.063	−.187	.009
L2 grit → FLLE	.470	.058	.540	< .001
FLA × L2 grit → FLLE	−.181	.075	−.132	.015
Gender → FLLE	−.041	.067	−.035	.544

Note. N = 285. PPCT = perceived psychologically controlling teaching; FLA = foreign language anxiety; FLLE = foreign language learning engagement. Gender was included as an observed exogenous covariate and was allowed to covary with PPCT, L2 grit, and the latent FLA × L2 grit interaction factor.

Section S4. Psychological network analysis

The seven-node network comprised observed mean scores for PPCT, FLA, L2 grit, and the four FLLE dimensions. PPCT was computed as the mean of its seven items, FLA as the mean of the eight retained items, L2 grit as the mean of its nine items, and behavioral, cognitive, emotional, and agentic engagement as the means of FLLE Items 1–5, 6–13, 14–17, and 18–22, respectively. The node scores were not standardized prior to estimation.

Among the 285 retained questionnaires, 124 of 13,110 focal item responses were missing (0.95%). These sporadic item-level missing responses were addressed using person-mean imputation within the corresponding scale before composite and network-node scores were computed. Consequently, no missing values remained in the seven network variables entered into the PNA. The `missing = "listwise"` specification was retained as a safeguard but did not result in any additional case deletion. The primary network was estimated from Pearson correlations using EBICglasso with the EBIC tuning parameter set to $\gamma = .50$. Edges represent regularized partial correlations.

Edge-weight accuracy was evaluated with 5,000 nonparametric bootstrap samples. Centrality stability was evaluated with 5,000 case-dropping bootstrap samples. Expected influence was designated as the primary centrality index because it retains the sign of positive and negative edges. Sensitivity analyses re-estimated the network with $\gamma = .25$ and $\gamma = 0$.

Table S4. Zero-order Pearson correlations among the seven network nodes

Variable	PPCT	FLA	GRIT	BE	CE	EE	AE
PPCT	1.000	.303	−.255	−.342	−.261	−.216	−.307
FLA	.303	1.000	−.488	−.346	−.466	−.395	−.360
GRIT	−.255	−.488	1.000	.562	.533	.569	.526
BE	−.342	−.346	.562	1.000	.644	.676	.724
CE	−.261	−.466	.533	.644	1.000	.802	.750
EE	−.216	−.395	.569	.676	.802	1.000	.753
AE	−.307	−.360	.526	.724	.750	.753	1.000

Note. PPCT = perceived psychologically controlling teaching; FLA = foreign language anxiety; GRIT = L2 grit; BE = behavioral engagement; CE = cognitive engagement; EE = emotional engagement; AE = agentic engagement. Values are zero-order Pearson correlations among the observed mean scores entered into the psychological network analysis.

Table S5. Regularized partial-correlation edge weights in the primary seven-node network (γ = .50)

Node 1	Node 2	Weight	Direction	Retained
CE	EE	.459	Positive	Yes
BE	AE	.347	Positive	Yes
CE	AE	.292	Positive	Yes
FLA	GRIT	−.273	Negative	Yes
EE	AE	.255	Positive	Yes
GRIT	BE	.207	Positive	Yes
FLA	CE	−.170	Negative	Yes
GRIT	EE	.159	Positive	Yes
BE	EE	.157	Positive	Yes
PPCT	FLA	.156	Positive	Yes
PPCT	BE	−.137	Negative	Yes
BE	CE	.070	Positive	Yes
PPCT	AE	−.049	Negative	Yes
GRIT	AE	.038	Positive	Yes
GRIT	CE	.032	Positive	Yes
PPCT	GRIT	−.003	Negative	Yes
FLA	BE	.000	Zero	No
PPCT	CE	.000	Zero	No
PPCT	EE	.000	Zero	No
FLA	EE	.000	Zero	No
FLA	AE	.000	Zero	No

Note. Weights are EBICglasso-regularized partial correlations. Zero-valued edges were not retained in the estimated network. Rows are ordered by absolute edge magnitude.

Table S6. Expected influence scores for the seven network nodes

Rank	Node	Expected influence
1	EE	1.030
2	AE	.883
3	CE	.683
4	BE	.644
5	GRIT	.161
6	PPCT	−.033
7	FLA	−.287

Note. Expected influence is the sum of signed edge weights connected to each node. Larger positive values indicate stronger net positive connectivity; negative values indicate that negative connections predominate.

Table S7. Correlation-stability coefficients for centrality indices

Centrality index	CS coefficient	Use in interpretation
Expected influence	.751	Primary index; interpreted
Strength	.751	Reported for transparency; not emphasized
Closeness	.361	Not interpreted
Betweenness	.049	Not interpreted

Note. CS = correlation-stability coefficient. Substantive interpretation in the main text focused on expected influence.

Table S8. Sensitivity of the estimated network to the EBIC tuning parameter

γ	Nonzero edges	Positive	Negative	Difference from primary network
.50 (primary)	16	11	5	Reference network
.25	16	11	5	Identical to the primary network
0	17	12	5	One additional weak positive PPCT–EE edge (weight = .031)

Note. At γ = .25, all edge weights and retained-edge decisions were identical to the primary γ = .50 network. At γ = 0, all primary edges retained their directions and only the weak PPCT–EE edge was added.

Figure S1. Accuracy of the edge-weight estimates. Black points represent bootstrap means, red points represent sample estimates, and horizontal lines represent 95% bootstrap confidence intervals. Wider intervals indicate greater estimation uncertainty.

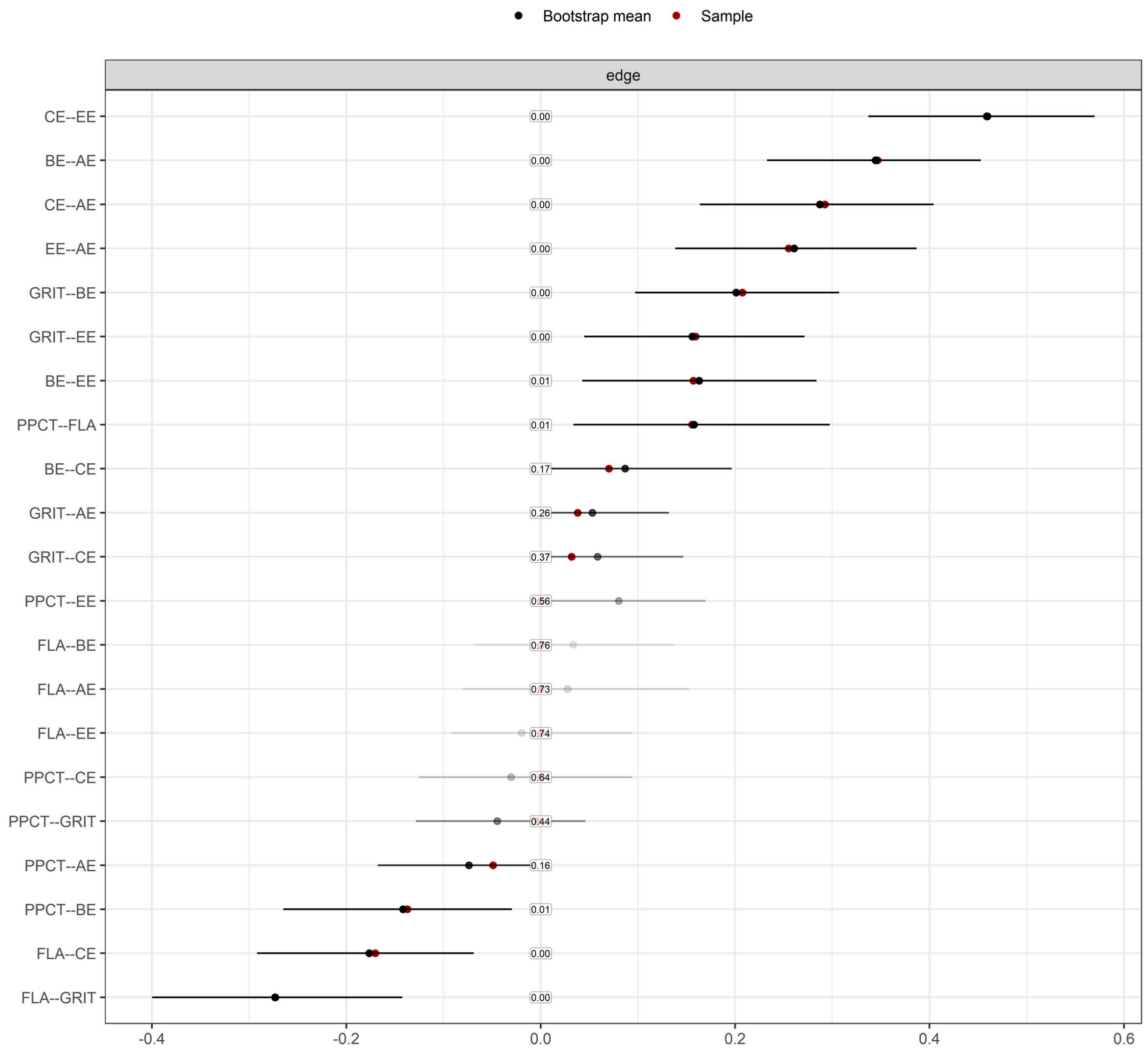

Figure S2. Bootstrapped edge-weight difference test. Colored diagonal cells represent the sample edge weights. Black cells indicate statistically significant differences between pairs of edge weights at the .05 level; gray cells indicate nonsignificant differences.

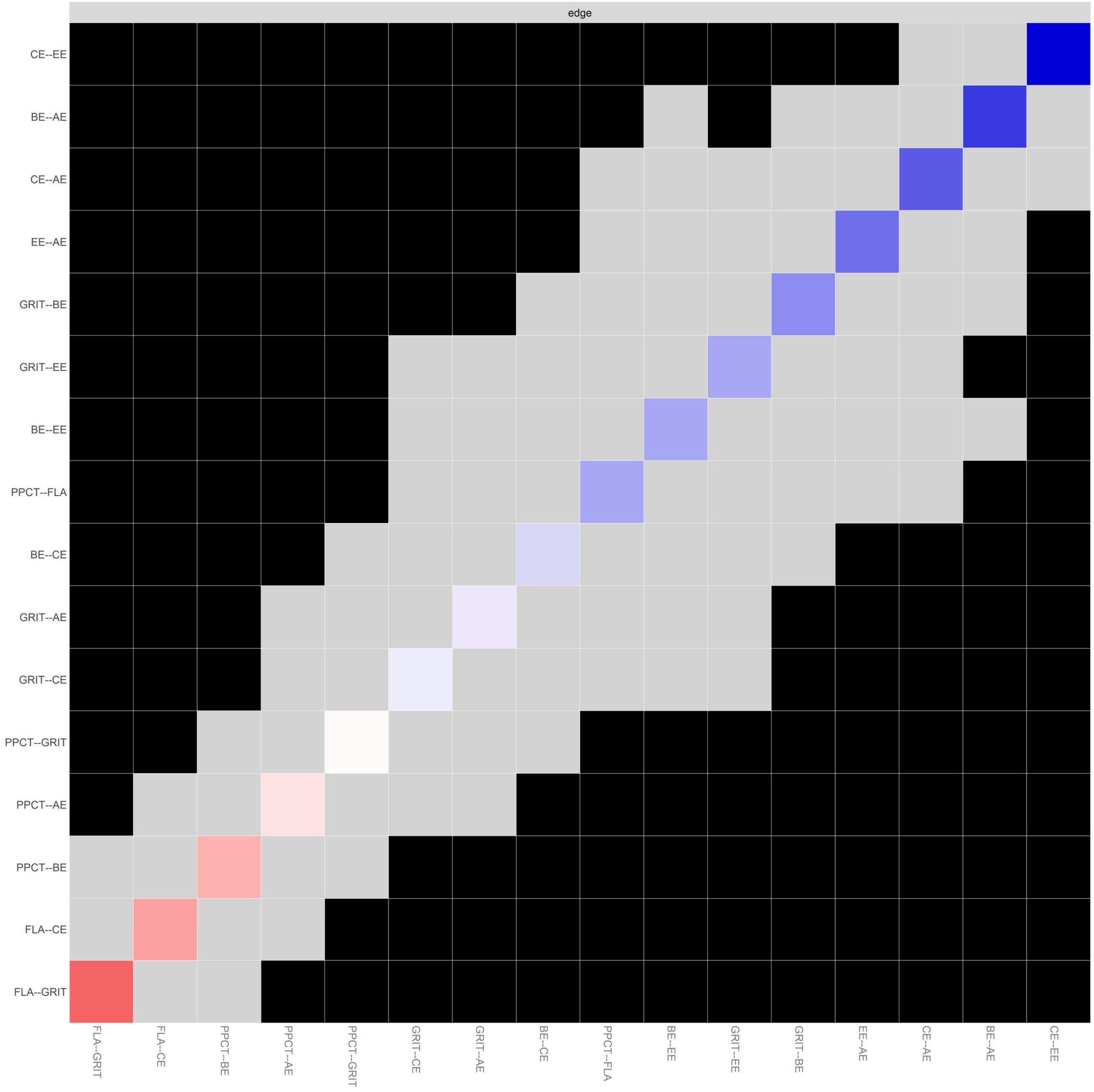

Figure S3. Case-dropping bootstrap stability of expected influence. The line represents the average correlation between expected-influence estimates from case-dropped subsets and those from the original sample; the shaded area represents the 95% confidence interval. The correlation-stability coefficient for expected influence was .751.

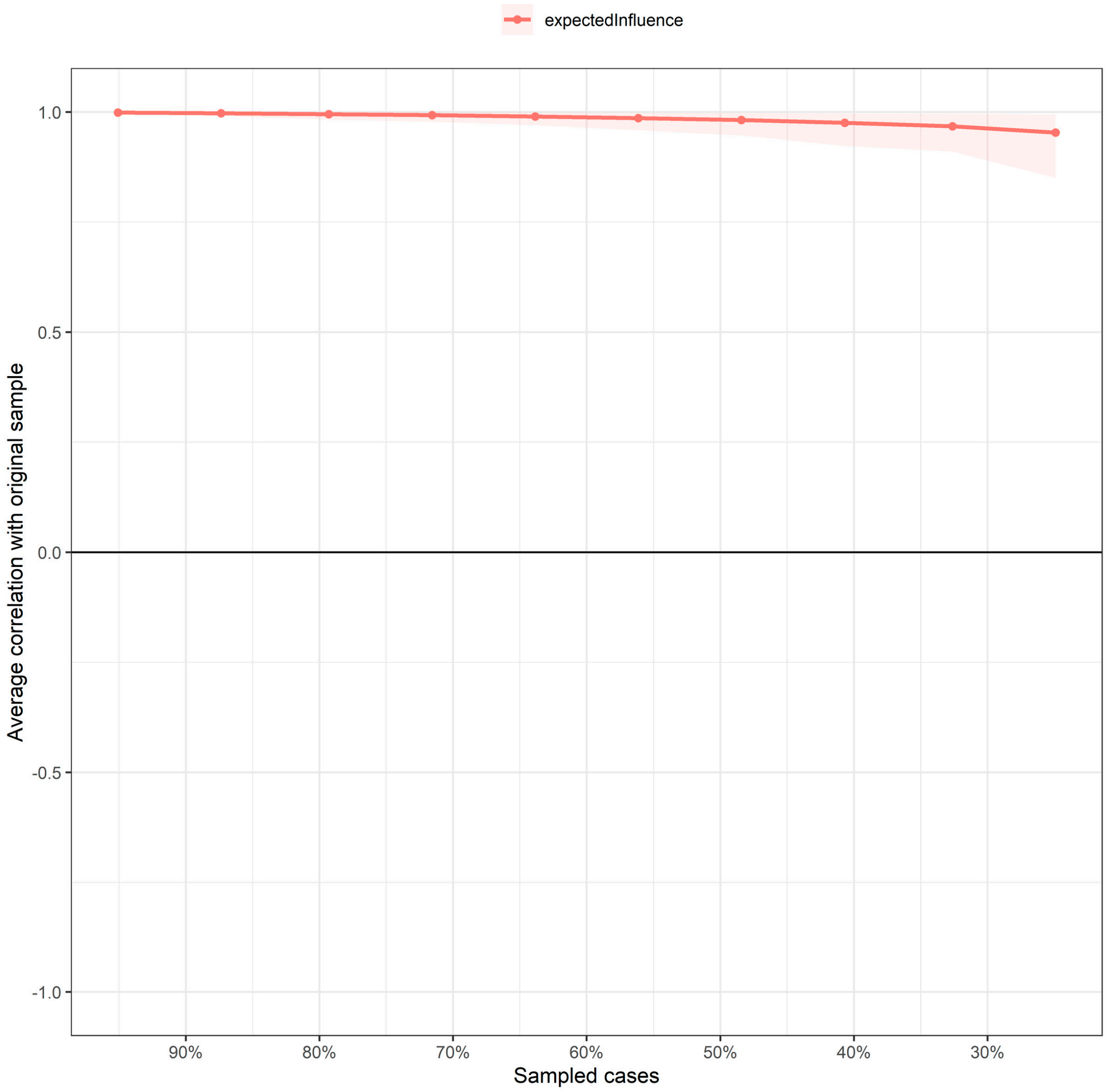

Supplementary Note S1. Key R syntax for psychological network analysis

```
analysis_data <- scores[complete.cases(scores), ]

network_est <- estimateNetwork(
  analysis_data,
  default = "EBICglasso",
  tuning = 0.50,
  corMethod = "cor",
  missing = "listwise"
)

bootstrap_nonparametric <- bootnet(
  network_est,
  nBoots = 5000,
  type = "nonparametric",
  statistics = c(
    "edge",
    "strength",
    "closeness",
    "betweenness",
    "expectedInfluence"
  )
)

bootstrap_stability <- bootnet(
  network_est,
  nBoots = 5000,
  type = "case",
  statistics = c(
    "strength",
    "closeness",
    "betweenness",
    "expectedInfluence"
  )
)

corStability(
  bootstrap_stability,
  statistics = c(
    "strength",
    "closeness",
    "betweenness",
    "expectedInfluence"
  )
)

network_gamma025 <- estimateNetwork(
  analysis_data,
  default = "EBICglasso",
```

```
  tuning = 0.25,
  corMethod = "cor",
  missing = "listwise"
)

network_gamma000 <- estimateNetwork(
  analysis_data,
  default = "EBICglasso",
  tuning = 0.00,
  corMethod = "cor",
  missing = "listwise"
)
```

Section S5. Item-level measurement and sensitivity analyses of L2 grit

Because the primary structural model represented L2 grit using item parcels, additional analyses were conducted to examine whether the central moderation finding depended on this representation. The original L2 grit parcels were constructed using an item-to-construct balancing approach based on the item factor loadings. GroupW1 comprised Items 1, 4, and 8 (F1, F4, and F8); GroupW2 comprised Items 3, 5, and 7 (F3, F5, and F7); and GroupW3 comprised Items 2, 6, and 9 (F2, F6, and F9). Item-level confirmatory factor analyses were first performed using the original L2 grit items. Given the five-point Likert response format, items were treated as ordered categorical indicators and models were estimated using diagonally weighted least squares (DWLS).

The original nine-item one-factor model showed poor fit, $\chi^2(27) = 738$, $p < .001$, CFI = .933, TLI = .911, RMSEA = .304, 95% CI [.286, .324], and SRMR = .157. Standardized factor loadings ranged from −.117 to .942. These results indicate that the nine items were not adequately represented as a homogeneous unidimensional construct at the item level.

A theoretically differentiated structure separating perseverance of effort from consistency of interest was subsequently examined. Item 2 showed anomalous behavior in the present sample. In the nine-item one-factor model, Item 2 was the only indicator with a negative standardized loading ($\beta = -.117$), whereas the remaining items loaded positively on the factor. Item 2 also showed an inconsistent association with the remaining consistency-of-interest indicators and substantially destabilized estimation when included in the ordinal two-factor model. Accordingly, Item 2 was excluded only from the alternative dimension-specific representation used in the sensitivity analyses.

The alternative representation included five perseverance-of-effort items (Items 1, 3, 5, 6, and 9) and three consistency-of-interest items (Items 4, 7, and 8). Because F1 and F3 and F5 and F6 were highly similar in wording and content, their residuals were allowed to correlate. The resulting model yielded $\chi^2(17) = 121$, $p < .001$, CFI = .990, TLI = .984, RMSEA = .147, and SRMR = .079. Standardized factor loadings ranged from .554 to .931. Perseverance of effort and consistency of interest were moderately correlated, $r = .553$, $p < .001$. Although RMSEA remained elevated, the retained items showed a substantially clearer dimension-specific structure than the original unidimensional representation.

To examine whether the moderation result depended on the original parcel-based general L2 grit specification, dimension-specific sensitivity analyses were conducted using PROCESS Model 14. Perseverance of effort and consistency of interest were entered separately as moderators of the association between foreign language anxiety and foreign language learning engagement. Gender was included as a covariate. Bootstrap confidence intervals were based on 5,000 resamples.

Table S9. Item-level CFA results for L2 grit

Representation	Dimension	Item	Standardized loading
Nine-item one-factor	General L2 grit	F1	.942
		F2	−.117
		F3	.936
		F4	.612
		F5	.678
		F6	.788
		F7	.509
		F8	.683
		F9	.607
Eight-item two-factor	Perseverance of effort	F1	.863
		F3	.852
		F5	.554
		F6	.746
		F9	.695
	Consistency of interest	F4	.798
		F7	.661
		F8	.931

Note. Items were treated as ordered categorical indicators and estimated using DWLS. The nine-item one-factor model showed $\chi^2(27) = 738$, $p < .001$, CFI = .933, TLI = .911, RMSEA = .304, 95% CI [.286, .324], and SRMR = .157. In the eight-item two-factor model, residual covariances were specified between F1 and F3 (standardized residual covariance = .728) and between F5 and F6 (.597) because of substantial overlap in item wording and content. The two dimensions correlated at $r = .553$. Model fit for the eight-item representation was $\chi^2(17) = 121$, $p < .001$, CFI = .990, TLI = .984, RMSEA = .147, and SRMR = .079. Item F2 was excluded only from the alternative sensitivity representation because of its anomalous loading pattern and estimation instability.

Table S10. Gender-adjusted dimension-specific moderated indirect-association models

Panel A. Regression coefficients

Outcome	Predictor	Effort model b	SE	p	Interest model b	SE	p
FLA	PPCT	.443	.070	< .001	.443	.070	< .001
FLA	Gender	.363	.094	< .001	.363	.094	< .001
FLLE	PPCT	−.175	.056	.002	−.178	.063	.005
FLLE	FLA	.160	.130	.219	.265	.206	.198
FLLE	Grit dimension	.739	.120	< .001	.619	.161	< .001
FLLE	FLA × grit dimension	−.114	.040	.005	−.135	.049	.007
FLLE	Gender	.017	.072	.811	−.055	.080	.496

Panel B. Interaction and moderated indirect association

Moderator	ΔR^2 for interaction	p	Index of moderated mediation	BootSE	BootLLCI	BootULCI
Perseverance of effort	.0166	.0048	−.0505	.0223	−.0996	−.0128
Consistency of interest	.0190	.0066	−.0597	.0286	−.1218	−.0085

Panel C. Conditional FLA–FLLE associations

Moderator	Level	Conditional effect	SE	p	95% CI
Effort	Low (2.20)	−.091	.058	.121	[−.205, .024]
	Median (2.80)	−.159	.049	.001	[−.255, −.063]
	High (3.80)	−.273	.058	< .001	[−.386, −.160]
Interest	Low (3.00)	−.139	.073	.060	[−.283, .006]
	Median (4.00)	−.274	.053	< .001	[−.378, −.169]
	High (4.33)	−.319	.055	< .001	[−.427, −.210]

Note. PROCESS Model 14 was estimated separately for perseverance of effort and consistency of interest. PPCT = perceived psychologically controlling teaching; FLA = foreign language anxiety; FLLE = foreign language learning engagement. Gender was entered as a covariate in both equations. Conditional values correspond to the 16th, 50th, and 84th percentiles of the moderator. Bootstrap confidence intervals were based on 5,000 resamples. These percentile-based probing values were used only in these dimension-specific sensitivity analyses and differ from the mean ± 1 SD probing used in the primary latent model.

Bifactor sensitivity analysis

Following Liu et al. (2022), an additional bifactor CFA was estimated in which all nine items loaded on a general L2 grit factor and simultaneously on their respective perseverance-of-effort or consistency-of-interest specific factors, with the three latent factors constrained to be orthogonal. Using DWLS with the ordinal item responses, the model showed excellent global fit, $\chi^2(18) = 13.70$, $p = .747$, CFI = 1.000, TLI = 1.001, RMSEA = .000, 95% CI [.000, .038], and SRMR = .027. However, the solution was inadmissible: Item 6 had a negative residual variance (−.257), the perseverance-specific factor had mixed specific loadings and a nonsignificant variance (.037, $p = .276$), and Item 2 retained a negative loading on the general factor ($\beta = -.224$).

Because Liu et al. (2022) used robust maximum-likelihood estimation, the same bifactor specification was re-estimated using robust ML as an estimator sensitivity check. Global fit again appeared good, scaled $\chi^2(18) = 26.50$, $p = .089$, CFI = .990, TLI = .980, robust RMSEA = .044, 95% CI [.000, .078], and SRMR = .023. The substantive irregularities persisted: Item 6 again had a negative residual variance (−.236), the perseverance-specific factor remained weakly defined (variance = .075, $p = .191$), and Item 2 again loaded negatively on the general factor ($\beta = -.233$). Thus, favorable global fit did not yield a stable admissible bifactor solution in the present sample.

Table S10A. Selected results from the L2 grit bifactor sensitivity analyses

**Index / parameter**	**DWLS**	**Robust ML**
χ^2 (df = 18)	13.70	26.50 (scaled)
p	.747	.089
CFI	1.000	.990
TLI	1.001	.980
RMSEA	.000	.044 (robust)
RMSEA 95% CI	[.000, .038]	[.000, .078]
SRMR	.027	.023
Item 2 → general grit, β	−.224	−.233
Item 6 residual variance	−.257	−.236
Perseverance-specific variance	.037, p = .276	.075, p = .191

Note. The bifactor specification included a general L2 grit factor and orthogonal perseverance-of-effort and consistency-of-interest specific factors. Negative residual variances indicate an improper solution. The robust-ML column reports the scaled chi-square and robust RMSEA.

Sensitivity analysis excluding Item 2

Because Item 2 showed an anomalous negative loading on the general factor, an additional sensitivity analysis examined whether the primary moderation result depended on its inclusion. Item 2 was removed, an eight-item general-grit mean score was computed from the remaining items, and PROCESS Model 14 was re-estimated with PPCT as X, FLA as M, the eight-item grit score as W, and aggregate FLLE as Y. Percentile bootstrap confidence intervals were based on 5,000 resamples.

The FLA × grit interaction remained significant, $b = -.196$, $SE = .061$, $p = .0014$, 95% CI [−.316, −.076], $\Delta R^2 = .021$. The index of moderated indirect association was −.0478 (BootSE = .0190), 95% bootstrap CI [−.0903, −.0154]. The conditional indirect association was not different from zero at the lower level of grit ($b = -.0122$, 95% bootstrap CI [−.0594, .0337]) or at the median level ($b = -.0361$, 95% bootstrap CI [−.0785, .0004]), but differed from zero at the higher level ($b = -.0734$, 95% bootstrap CI [−.1256, −.0341]). Removing Item 2 therefore did not materially alter the direction or substantive interpretation of the moderation pattern.

Table S10B. Moderated indirect-association sensitivity analysis using the eight-item grit score

**Effect**	**Estimate**	**SE / BootSE**	**p**	**95% CI**
FLA × grit	−.1960	.0608	.0014	[−.3156, −.0764]
Conditional FLA effect: low grit	−.0500	.0802	.534	[−.2079, .1080]
Conditional FLA effect: median grit	−.1480	.0677	.0297	[−.2812, −.0147]
Conditional FLA effect: high grit	−.3009	.0734	.0001	[−.4453, −.1565]
Indirect association: low grit	−.0122	.0229	—	[−.0594, .0337]
Indirect association: median grit	−.0361	.0196	—	[−.0785, .0004]
Indirect association: high grit	−.0734	.0229	—	[−.1256, −.0341]
Index of moderated indirect association	−.0478	.0190	—	[−.0903, −.0154]

Note. Bootstrap confidence intervals were based on 5,000 percentile bootstrap samples. Low, median, and high grit correspond to the 16th, 50th, and 84th percentiles of the eight-item grit score, respectively.

Taken together, the item-level analyses did not support a single unequivocal latent representation of L2 grit in the present sample. However, the negative FLA × grit interaction and the moderated indirect association were reproduced across alternative grit operationalizations, including perseverance of effort, consistency of interest, and an eight-item general-grit score excluding Item 2. These sensitivity analyses therefore support the substantive robustness of the moderation pattern while leaving the precise latent structure of L2 grit uncertain.

Section S6. Item-level confirmatory factor analyses of foreign language learning engagement

Additional item-level CFA was conducted to evaluate the multidimensional structure of FLLE. All 22 items were treated as ordered categorical indicators and estimated using DWLS.

A one-factor model in which all 22 items loaded on a single FLLE factor yielded $\chi^2(209) = 4450$, $p < .001$, CFI = .964, TLI = .960, RMSEA = .267, and SRMR = .126. Standardized factor loadings ranged from .631 to .971.

A correlated four-factor model corresponding to behavioral engagement, cognitive engagement, emotional engagement, and agentic engagement provided a substantially improved representation of the data, $\chi^2(203) = 2125$, $p < .001$, CFI = .984, TLI = .981, RMSEA = .183, and SRMR = .091. Standardized item loadings ranged from .675 to .994. Inter-factor correlations ranged from .643 to .902, indicating substantial shared variance while retaining a theoretically meaningful multidimensional structure.

A simple higher-order model was subsequently estimated in which the four first-order engagement dimensions loaded on a general FLLE factor. Although CFI (.982) and TLI (.980) remained high, the model produced an improper solution. The standardized loading of emotional engagement on the higher-order FLLE factor was 1.009, and the residual variance of emotional engagement was negative (−.013). Accordingly, the higher-order model was not interpreted as providing adequate empirical support for a strict general-factor representation of FLLE.

These results were more consistent with retaining the four engagement dimensions as analytically meaningful components than with treating the 22 items as a homogeneous single factor. In the primary SEM, FLLE was therefore interpreted as a pragmatic aggregate construct-level representation rather than as a psychometrically confirmed higher-order factor.

Table S11. Comparison of alternative item-level measurement models for FLLE

Model	χ^2	df	CFI	TLI	RMSEA	SRMR	Interpretation
One-factor FLLE	4450	209	.964	.960	.267	.126	Poor absolute fit
Correlated four-factor	2125	203	.984	.981	.183	.091	Substantially improved; multidimensional structure favored
Higher-order FLLE	2248	205	.982	.980	.187	.093	Improper solution

Note. Models were estimated using DWLS with all 22 FLLE items treated as ordered categorical indicators. The higher-order model produced an improper solution because the standardized loading of emotional engagement on the higher-order factor exceeded 1.00 (β = 1.009) and the residual variance of emotional engagement was negative.

Table S12. Dimension-specific sensitivity analyses of the moderated indirect association

Outcome	FLA × L2 grit b	SE	p	ΔR²	Index of moderated mediation	Boot 95% CI	Holm-adjusted p
Behavioral engagement	−.095	.054	.080	.007	−.042	[−.094, .001]	.084
Cognitive engagement	−.161	.073	.028	.012	−.071	[−.150, .006]	.084
Emotional engagement	−.184	.066	.006	.019	−.081	[−.156, −.019]	.024
Agentic engagement	−.125	.060	.039	.011	−.056	[−.108, −.006]	.084

Note. Each analysis used PROCESS Model 14 with PPCT as the predictor, FLA as the mediator, overall L2 grit as the moderator of the FLA–engagement association, and gender as a covariate. These analyses were treated as exploratory sensitivity analyses. Holm adjustment was applied across the four dimension-specific FLA × L2 grit interaction tests; the adjusted p values are shown in the final column. Bootstrap confidence intervals for the indices of moderated indirect association were based on 5,000 resamples and were interpreted descriptively rather than as a separately adjusted confirmatory test family.
